# A robust convolutional neural network for lung nodule detection in the presence of foreign bodies

**DOI:** 10.1038/s41598-020-69789-z

**Published:** 2020-07-31

**Authors:** Manuel Schultheiss, Sebastian A. Schober, Marie Lodde, Jannis Bodden, Juliane Aichele, Christina Müller-Leisse, Bernhard Renger, Franz Pfeiffer, Daniela Pfeiffer

**Affiliations:** 1 Chair of Biomedical Physics, Department of Physics and Munich School of BioEngineering, Technical University of Munich, 85748 Garching, Germany; 2 Department of Diagnostic and Interventional Radiology, School of Medicine & Klinikum rechts der Isar, Technical University of Munich, 81675 München, Germany

**Keywords:** Computer science, Lung cancer

## Abstract

Lung cancer is a major cause of death worldwide. As early detection can improve outcome, regular screening is of great interest, especially for certain risk groups. Besides low-dose computed tomography, chest X-ray is a potential option for screening. Convolutional network (CNN) based computer aided diagnosis systems have proven their ability of identifying nodules in radiographies and thus may assist radiologists in clinical practice. Based on segmented pulmonary nodules, we trained a CNN based one-stage detector (RetinaNet) with 257 annotated radiographs and 154 additional radiographs from a public dataset. We compared the performance of the convolutional network with the performance of two radiologists by conducting a reader study with 75 cases. Furthermore, the potential use for screening on patient level and the impact of foreign bodies with respect to false-positive detections was investigated. For nodule location detection, the architecture achieved a performance of 43 true-positives, 26 false-positives and 22 false-negatives. In comparison, performance of the two readers was 42 ± 2 true-positives, 28 ± 0 false-positives and 23 ± 2 false-negatives. For the screening task, we retrieved a ROC AUC value of 0.87 for the reader study test set. We found the trained RetinaNet architecture to be only slightly prone to foreign bodies in terms of misclassifications: out of 59 additional radiographs containing foreign bodies, false-positives in two radiographs were falsely detected due to foreign bodies.

## Introduction

With about 1.7 million deaths in 2018, lung cancer is one of the most common causes of cancer death^1^. As an early diagnosis improves outcomes^2^, regular screening with imaging methods is beneficial. For screening, especially low-dose computed tomography (CT) shows promising results in order to reduce mortality^3^. While a regular clinical chest CT requires a dose of 4–18 mSv^4^, low-dose CT applies an effective dose of around 1.5 mSv^3^. Compared to a posteroanterior study of the chest, which requires around 0.02 mSv^4^, the applied dose is still significantly higher. Hence, due to wider availability and possible avoidance of radiation induced long term effects, chest X-ray (CXR) is a potential alternative to chest CT for lung cancer screening. However, interpretation of CXR images is often challenging, as small lung nodules can easily be missed. For successful lung cancer screening it is mandatory to keep the rate of false-negatives low.

False-positive cancer diagnoses on the other hand may lead to substantial psychological consequences in patients, such as changes in self-perception or anxiety, as investigated for colorectal cancer^5^. Thus, for successful lung cancer screening, keeping the rate of false-negatives and false-positives as low as possible is mandatory.

With the rise in computing power, deep-learning based computer-aided diagnosis (CAD) systems have gained interest in the research community. Only recently, performance of human readers in disciplines such as breast cancer screening^6^ and dermoscopic melanoma image classification^7, 8^ was met or even exceeded. For mammography and chest X-ray classification, networks which are trained with case-level labels showed promising results^9–12^. However, such systems can only provide disease locations by the use of techniques such as saliency maps^13^. As these usually provide only inaccurate location boundaries, it is of interest to train such system with detailed annotations such as box coordinates or segmentations. Besides, it is also possible to train such networks in a semi-supervised manner, e.g. where a part of the data is labeled on pixel-level and the remaining radiographs are annotated on case-level^14^. For deep learning applied on CXR images with pixel level annotations, U-Net-like architectures can be employed for segmentation tasks^15, 16^. Current state of the art methods for pneumothorax detection^17^ or mammography screenin^6^ make use of box-annotations, which can be derived from pixel wise annotations. Both aforementioned studies use a RetinaNet architecture, a one stage detector^18, 19^, which is characterized by a faster inference time than two stage detectors^20, 21^. The aim of this study was to train a RetinaNet detector for the task of pulmonary nodule detection, which is robust to foreign bodies. We evaluated its accuracy for screening and nodule detection tasks. Furthermore, we compared its performance to the participants of a reader study.

## Results

### Nodule location detection

**Figure 1:**
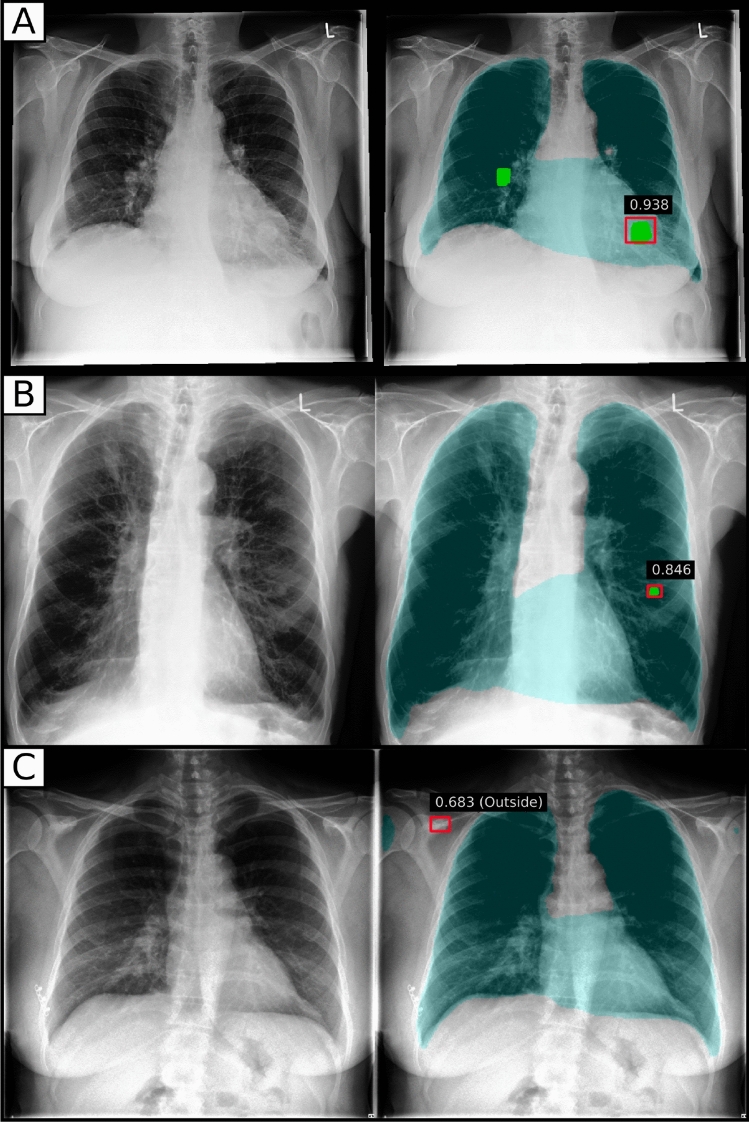
Chest radiographies with nodules detected by RetinaNet. The ground-truth is marked in green and predictions are indicated by red rectangles. Predicted lung segmentation masks are marked in cyan color. (**A**) True-positive prediction (0.938) marked with a red rectangle by the CNN and an undetected, false-negative nodule in the left lung lobe. (**B**) True-positive prediction within the right lung lobe. (**C**) False-positive prediction outside of the chest.

For nodule localisation, the assessed RetinaNet architecture achieved 43 true-positives, 26 false-positives and 22 false-negatives. In comparison, performance of the two readers was was 42 ± 2 true-positives, 28 ± 0 false-positives and 23 ± 2 false-negatives. Detailed results are shown in Table 1. If not otherwise stated, all results in this paper are given in the form mean ± standard deviation. The nodule detection performance of RetinaNet can be inspected visually (Fig. 1). Lung segmentation was used to exclude extrathoracic detections. For lung segmentation a Dice score of 0.97 was achieved.

**Table 1:** Results for the nodule detection task for radiologists and the RetinaNet model. Evaluation was performed with respect to true-positives (TP), false-positives (FP) and false-negatives (FN).

	TP	FP	FN
Radiologist A	40	28	25
Radiologist B	44	28	21
RetinaNet	43	26	22

**Figure 2:**
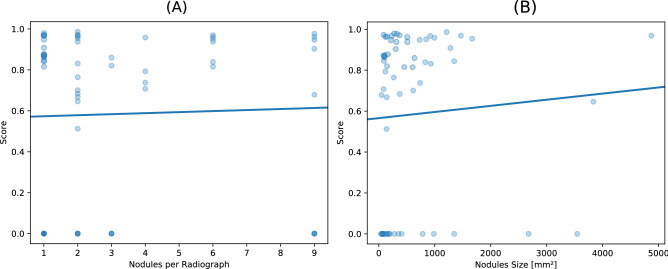
(**A**) Nodules per radiograph plotted against detection score with regression fit. (**B**) Nodule size plotted against detection score.

**Figure 3:**
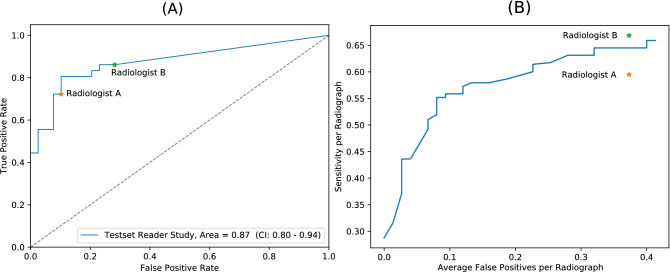
(**A**) ROC curve for the screening task. The blue diagonal line marks cases with an equal prediction score for healthy and unhealthy cases. (**B**) FROC curve plotting the average sensitivities per radiograph against the average number of false positives per radiograph.

In order to investigate if larger nodules can be detected more easily and if nodules in radiographs with many additional nodules are detectable more easily, we plotted these parameters against the detection score and performed a linear regression model fit for the number of nodules (Fig. 2A) and the nodule size (Fig. 2B). Furthermore, a free response receiver operating characteristic (FROC) curve is shown in Fig. 3B.

### Screening

The classification performance was also assessed on case-level using a ROC (receiver operating characteristic) curve. Here the true-positive rate is plotted over the false-positive rate. For RetinaNet, an AUC (area under the ROC curve) value of 0.87 with a confidence interval (CI) range from 0.80 to 0.94 was found for the model. The performance of the two radiologists for the case-level screening task was 26 TP/4 FP and 31 TP/11 FP respectively. The ROC curve for the model with radiologist scores is shown in Fig. 3A.

### Investigation of foreign body detections

**Figure 4:**
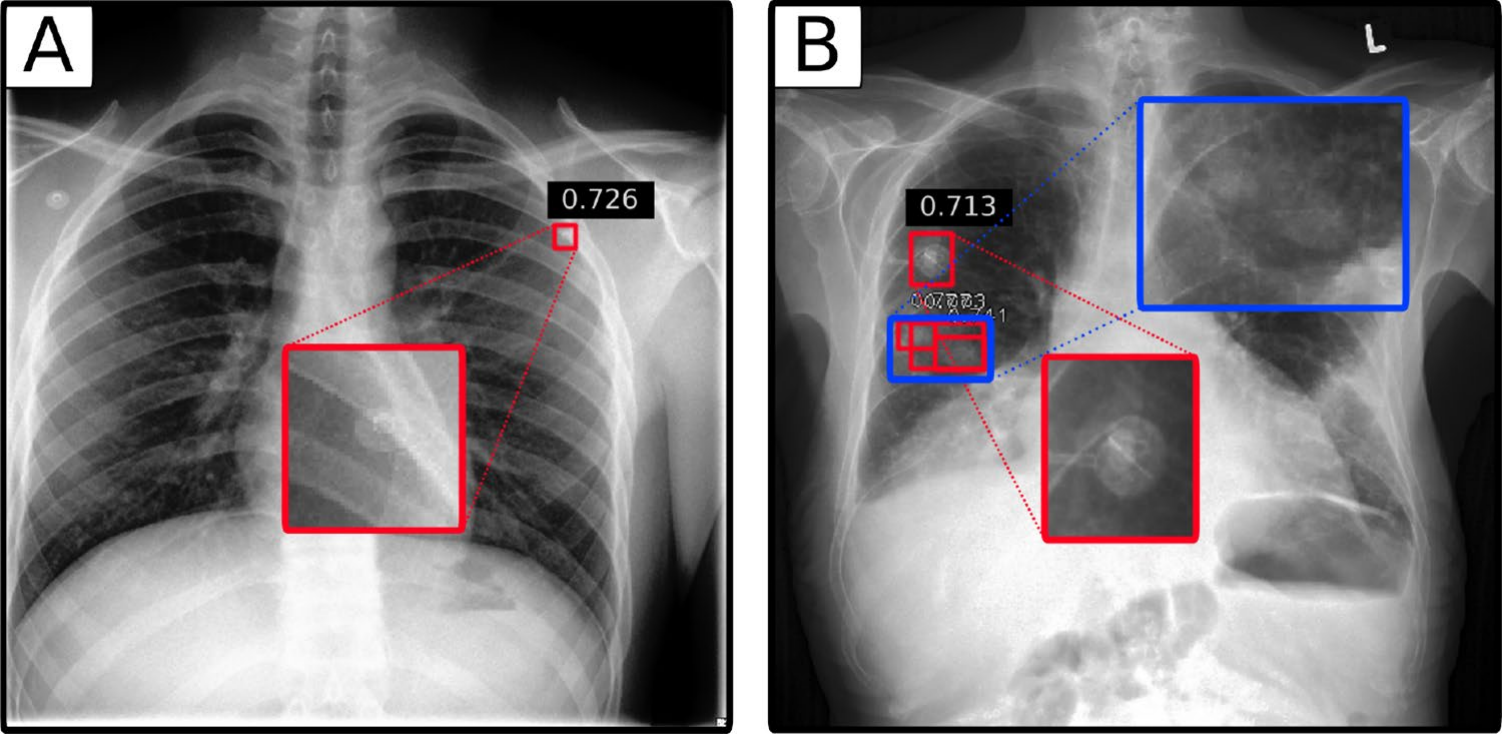
Chest radiographies with foreign bodies wrongly detected as pulmonary nodules. (**A**) ECG device electrode detected as nodule (false-positive). (**B**) Port detected as nodule (false-positive). Overlapping boxes resemble multiple detected nodes within a small area (blue box).

We investigated false-positive detections for five different types of foreign bodies (Table 2). These included ports, electrocardiography devices (ECG), surgical clips (Clips), sternum cerclages and pacemakers. For RetinaNet, one false-positive detection due to foreign bodies occurred for each, ECG and port. Examples of foreign body detections, as predicted by the RetinaNet architecture are shown in Fig. 4A and B. Overlapping boxes (Fig. 4B) occur rarely and probably due to a suspected bigger nodule behind two smaller ones.

**Table 2:** Number of radiographs with false-positive (FP) detections due to foreign bodies (FB) made by the RetinaNet architecture.

Foreign body	Total radiographs	Radiographs with FP due to FB
Port	26	1
ECG	21	1
Sternum cerclage	5	0
Clips	10	0
Pacemaker	3	0

False-positives due to FB only occurred for radiographs classified as port or ECG.

## Discussion

In this study, we trained and investigated a CNN for pulmonary nodule detection and compared the predictions made by the CNN to the results of two professional radiologists. Besides nodule localisation, the usability of the algorithm for case-level screening was investigated. An important aspect of this study was to evaluate the possibility of foreign bodies contributing to wrong decisions in CNN-based nodule detection systems. This was not investigated in previous work^9, 10, 22^ and therefore we evaluated the question, if foreign bodies are wrongly detected as nodules by a CNN.

For the nodule detection task, the CNN was able to outperform one radiologist. The CNN was found to identify larger nodules more easily, but also performed well on smaller nodules (Fig. 2B).

For the screening task, the underlying question was if a radiograph contains nodules or not. Case-level predictions were derived from the predicted box-annotations, similar as it was done for mammography classification by McKinney et al.^6^. Compared to CNNs already trained with case-level annotations^9^, CNNs trained with box-annotations, such as the investigated RetinaNet architecture, have substantial differences: Unfortunately, box-level annotations have to be generated additionally by an expert radiologist and cannot be retrieved easily from existing PACS data. While the availability of annotated data is a common limitation for the training of deep learning systems in a clinical setting, the investigated technology relies on the availability of such annotations. In case such annotations are not available, weakly-supervised training^23–25^ may be a possible alternative. However, in order to understand the CNNs decisions, CNNs trained with box-annotations allow a more detailed evaluation of retrieved results: CNNs trained with box annotations are capable of providing an independent score and an accurate location for each lesion. In contrast, CNNs trained with case level annotations can only provide a score for the whole image.

In literature, Wang et al.^9^ and Rajpurkar et al.^10^ reported a ROC AUC of 0.72 and 0.78 for weakly supervised CNN based lung nodule screening, respectively. For screening, we achieved a ROC AUC of 0.87 in our experiments.

Several studies have reported CAD performance for lung nodule detection. Here, sensitivities of CAD systems vary widely between literature, whereat a higher sensitivity usually yields a higher false-positive rate. Li et al.^26^ reported 47 of 66 nodules were correctly marked by a CAD system for CXR lung nodule detection. This gives a sensitivity of 0.71 at a mean false-positive rate of 1.3. For nodule detection Kim et al.^22^ reported a sensitivity of 0.83 at a false-positive rate per radiograph of 0.2. For CT based nodule detection CAD systems, an average sensitivity of 0.82 was reported at a cutoff of 3 false-positives per radiograph by Jacobs et al.^27^. At a false positive cutoff of about 0.2, their FROC curve yielded a sensitivity of around 0.53. To compare these results to our experiments, we can select a specific point on the FROC Curve (Fig. 3B), e.g. a sensitivity of 0.59 at a false-positive rate of 0.2 per radiograph.

Comparison of performance within literature is difficult, however, as results highly depend on the dataset (e.g. nodule count and nodule size) and objective of the study. Quekel et al.^28^ reported lesion miss rates for lung cancer on chest radiographs between 25 and 90% for different studies involving human observers. Therefore, a comparison of the given ROC and FROC results between different papers should be interpreted with caution.

Besides, the potential of deep learning systems, this study also shows that before clinical use of a deep learning system, it has to be carefully assessed how uncommon image characteristics can contribute to false decisions of CNNs. While we could have designed an ideal dataset without foreign bodies, a remarkable feature of our dataset is that we intentionally included foreign bodies in our training and test sets. Therefore, our dataset is closer to the clinical routine and tests the robustness of the detection algorithm. Hence, an important finding of this study was, that the trained CNN produced only a few false-positive nodule detections in radiographs due to foreign bodies (in two out of 59 radiographs). This low number should not be neglected, however: in clinical routine, a lot of patients have foreign bodies like ECG electrodes typically seen in inpatient treatment or ports in oncological patients with a history of chemotherapy.

The present study has some limitations: above all, the size of the dataset was rather small. While we used 411 radiographs for training, other studies use larger datasets (e.g. 11,734 images for training by Mckinney et al.^6^ for mammography), which could further improve performance. Next, we only utilized PA radiographies for the detection task. In addition, the use of additional lateral chest radiographs could increase the performance further, but requires additional segmentations from human experts.

Moreover, we only used a single center data-set from our institution, which may inhibit the ability to translate the model to different populations and devices^29^. Last, we limited the CNN input resolution to $512 \times 512$ pixels, in order to reduce the computational workload.

## Conclusion

In this study, we trained and evaluated a RetinaNet based CNN and conducted a reader study. In summary, the presented CNN has the potential to help radiologists during clinical routine and is robust to foreign bodies. The CNN’s decisions can be followed by inspection of individual lesion scores and box-predictions, which is an advantage over other CNN architectures.

As there are still a few foreign body detections, in future work it has to be investigated, if it is sufficient to train with a larger dataset, or an auxiliary CNN^30^ is needed to identify abnormal cases and react correspondingly. With advances in healthcare digitisation, information about foreign bodies may also be available in machine readable form soon. Such information, stored in patient records, may be used to alter the CNNs decision (e.g. to invalidate lesion scores in the region of a known pacemaker). Furthermore, to enhance classification performance, we plan to collect and annotate more data. Additionally, the CNN could be trained to detect multiple pathologies, as done for case-level annotations in prior studies^10^.

## Materials and methods

### Dataset

Data access was approved by the institutional ethics committee at Klinikum Rechts der Isar (Ethikvotum 87/18 S) and the data was anonymized. The ethics committee has waived the need for informed consent. All research was performed in accordance with relevant guidelines and regulations. A dataset of 391 CXRs (Chest PA) was collected from our institution’s picture archiving and communication system (PACS). Patient demographics for training and test sets are shown in Table 3. Additional clinical information from the medical report, such as follow-up CT scans were available to verify the diagnosis. Thereby, case-level ground-truth labels (unsuspicious or nodulous) were assigned based on the diagnosis of two radiologists: the first radiologist made the diagnosis in clinical routine and a second radiologist (JB, 3 years of experience in chest imaging) verified and segmented the nodules retrospectively using our in-house built web-based platform. For the reader study test-set, one more radiologist verified the segmented nodules (DP, 12 years of experience).

From the segmentations, bounding boxes were extracted based on the segmentation boundaries. From the radiographs with nodules, 257 were used for training. Training data was supplemented by the Japanese Society of Radiological Technology (JSRT) dataset^31^, from which 154 additional radiographs with annotated nodules were obtained. Therefore, the total number of radiographs used for training was 411.

Additionally, lung segmentations for all 247 JSRT files were obtained from the segmention in chest radiography (SCR) database^32^ in order to train a lung segmentation network. Please note that data for lung segmentation also includes 93 additional non-nodulous images from the JSRT database. For lung segmentation train, validation and test set size was set to 157, 40 and 50.

**Table 3:** Patient demographics for training and test subsets. Mass size is given as a fraction of the radiograph size (1.0 would indicate every pixel of the radiograph is a nodule). As for screening and foreign body (FB) test sets segmentations were unavailable, nodule mass and location were not provided. Within a radiograph multiple foreign bodies may occur. Secondary pathologies (SP) were excluded from the reader study. AM indicates acromastinum induced artifacts, which often show nodule-like morphological characteristics.

	Training	Test	
		Reader study	FB
Number	257	75	59
Age	64 ± 13.43	55 ± 15.93	55 ± 17.41
Left lobe	420	32	–
Right lobe	435	33	–
Mass size	0.003 ± 0.009	0.004 ± 0.006	–
Nodulous	137	20	–
Nodulous + SP	30	0	–
Nodulous + FB	90	16	26
Unsuspicious	–	20	–
Unsuspicious + SP	–	–	–
Unsuspicious + FB	–	16	33
Unsuspicious + AM	–	3	–
FB port	46	7	26
FB ECG	6	4	21
FB sternalcerclage	4	1	5
FB clips	15	4	10
FB pacemaker	3	1	3
FB other	24	20	0

**Figure 5:**
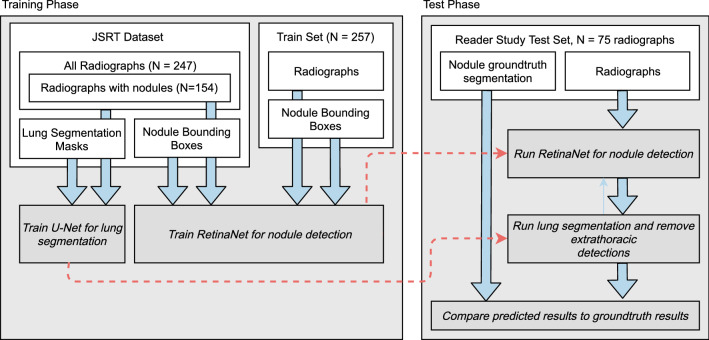
General workflow for training and test phase.

### Network training

General workflow for network training is illustrated in Fig. 5. For nodule detection, we employed a RetinaNet architecture^18^. This architecture was successfully utilitzed in prior literature^6, 22^ for nodule detection in radiographs. It inputs a preprocessed radiograph and outputs multiple box-coordinates of nodule locations with additional scores (assessing confidence).

For preprocessing, images were resampled to $512 \times 512$ pixels. Afterwards, histogram equalization was performed. Resulting intensities were normalized to values between 0 and 1.

Training was performed using a batch size of 1 with 1000 steps per epoch. For the utilized loss function (focal loss), hyperparameters were set to $\alpha = 0.25, \gamma = 2.0$. The initial learning rate was set to $10^{-5}$ and reduced by factor 0.1 after 3 epochs of stagnating loss ($\delta = 0.0001$). The network was trained for 50 epochs in total. Data augmentation transformations included contrast, brightness, shear, scale, flip, and translation. From the training set, 80 percent of the radiographs were used for training and 20 percent for validation. None of the training or validation data was part of the reader study or foreign body test sets. Models were implemented based on *keras-retinanet*^33^ using Tensorflow^34^ and Keras^35^. As a RetinaNet backbone, ResNet-101^36^ was used.

**Figure 6:**
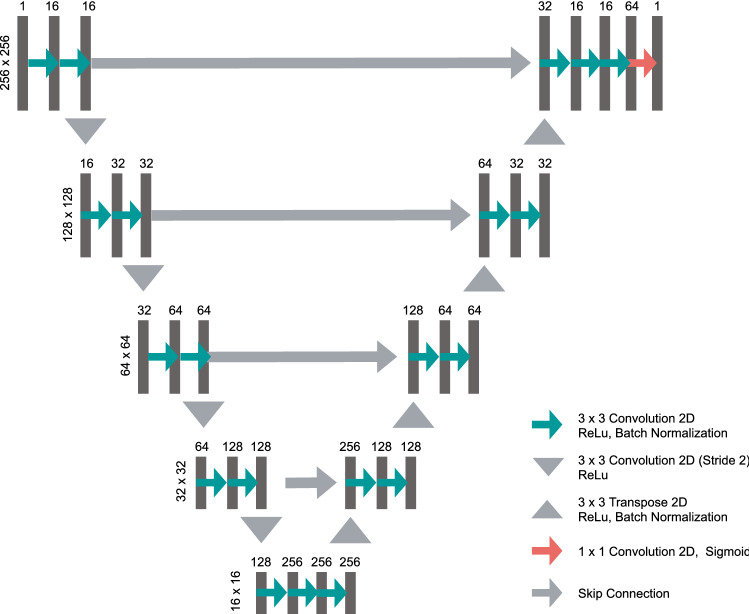
Utilized U-Net architecture for lung segmentation.

To invalidate extrathoracic nodule detections made by RetinaNet, an additional lung segmentation network was developed. For lung segmentation, an U-Net^15^ like architecture is applied in illustrated in Fig. 6. U-Net like architectures were successfully applied for lung segmentation in previous literature^16^. Training masks were generated by combining the left lung lob, right lung lobe and heart mask from the SCR dataset. An Adam optimizer with a learning rate of $10^{-4}$ was used. Total number of epochs was set to 30. Augmentation operations included zoom, height, shift and rotation. As a loss function, the Dice loss according to^37^ was used.

### Metrics

In each radiograph, we evaluated the number of true-positives (TP), false-positives (FP) and false-negatives (FN). True-negatives were not counted, as these include all possible remaining boxes within the radiograph. To determine the aforementioned numbers, a distance measurement is required, whereat we utilized a method similar to Shapira et al.^25^: within a single radiograph, first the center of masses of all ground-truth and prediction lesions are determined. Next, between each pair $(G_i,P_i)$ of a ground-truth lesion $G_i$ and a prediction lesion $P_i$, the euclidean distance is calculated. If the euclidean distance is below a certain threshold D, the nodule accounts as a true-positive. If there is no neighbour within the distance D for a $G_i$ or $P_i$, the nodule counts as false-negative and false-positive respectively. For a 512 × 512 pixel image we set the value of D to 23, which means a distance below 23 pixels for lesion centers between ground-truth and prediction yields a TP. To determine this value, we let the radiologist who annotated the groundtruth mark the lesion center in an additional experiment. Afterwards we calculated the distances between groundtruth segmentation center-of-mass and marked lesion center. The maximum distance between the groundtruth center-of-mass and the radiologist mark yielded 23 pixels. Furthermore, the sensitivity of the lesion detection can be controlled by ignoring predictions below a certain lesion score. Setting a lower threshold usually increases sensitivity, but also false-positives. This trend was visualized for different thresholds using a FROC curve, similar to Kim et al.^22^. For the absolute true-positive and false-positive numbers for RetinaNet results, we set a threshold of 0.6, which yields a lower false-positive rate than radiologists and therefore makes the absolute number of true-positives comparable. For evaluation of the nodule location detection task, the returned boxes were analyzed with respect to ground-truth annotations using the described metric.

Additionally it is required to retrieve a case-level score, for the screening task. This case-level score indicates whether there is one or more nodule in the radiograph. As we retrieved individual nodule scores from the RetinaNet predictions, we chose the maximum of all nodule scores within the radiograph as a case-level score.

### Reader study setup

For the reader study, two radiologists (CML and JA) interpreted 75 chest PA (posterior anterior) radiographs. The radiologists had 4 and 6 years experience. In order to simulate a clinical setting, each radiologist was given a time constraint of 10 seconds per radiograph. The assignment was to mark all nodules with a mouse click using our in-house built web-based platform. At least one nodule occured in 36 radiographs. Total nodule count was 65 and the average nodule count in radiographs with nodules was 1.8 ± 1.6.

### Statistical analysis

The bootstrap approach^38^ was used to calculate CIs of the ROC retrieved in the screening task for the RetinaNet architecture. We conducted the following experiment with 1,000 replications: In each experiment, we selected 75 random samples from the test set and calculated the ROC AUC values from these samples. In order to retrieve the 95% confidence interval, we sorted the resulting AUCs from all experiments incrementally and took the AUC value at 2.5% and 97.5% as minimum and maximum of the CI, respectively.

## Data Availability

Models for inference can be retrieved from the authors on reasonable request. The data for training is not available due to patient privacy. However, all methods are described in sufficient detail in order to be replicated with own data.

## References

[1] Bray F (2018). Global cancer statistics 2018: GLOBOCAN estimates of incidence and mortality worldwide for 36 cancers in 185 countries. Cancer J. Clin..

[2] Yang P (2009). Epidemiology of lung cancer prognosis: quantity and quality of life. Cancer.

[3] The National Lung Screening Trial Research Team. Reduced lung-cancer mortality with low-dose computed tomographic screening. *N. Engl. J. Med.* **365**, 395–409. 10.1056/NEJMoa1102873 (2011). 10.1056/NEJMoa1102873 PMC4356534 21714641

[4] Mettler FA, Huda W, Yoshizumi TT, Mahesh M (2008). Effective doses in radiology and diagnostic nuclear medicine: a catalog. Radiology.

[5] Toft EL, Kaae SE, Malmqvist J, Brodersen J (2019). Psychosocial consequences of receiving false-positive colorectal cancer screening results: a qualitative study. Scand. J. Prim. Health Care.

[6] Mckinney, S. M. *et al.* International evaluation of an AI system for breast cancer screening. *Nature* **577**, 10.1038/s41586-019-1799-6 (2020). 10.1038/s41586-019-1799-6 31894144

[7] Brinker TJ (2019). Deep learning outperformed 136 of 157 dermatologists in a head-to-head dermoscopic melanoma image classification task. Eur. J. Cancer.

[8] Esteva A (2017). Dermatologist-level classification of skin cancer with deep neural networks. Nature.

[9] Wang, X. *et al.* Chest X-ray8: hospital-scale chest X-ray database and benchmarks on weakly-supervised classification and localization of common thorax diseases. *Proceedings: 30th IEEE Conference on Computer Vision and Pattern Recognition, CVPR 2017* 2017, 3462–3471, 10.1109/CVPR.2017.369 (2017). arXiv:1705.02315.

[10] Rajpurkar, P. *et al.* CheXNet: radiologist-level pneumonia detection on chest X-rays with deep learning. 3–9, 1711.05225 (2017). arXiv:1711.05225.

[11] Ausawalaithong, W., Marukatat, S., Thirach, A. & Wilaiprasitporn, T. Automatic lung cancer prediction from chest X-ray images using deep learning approach. (2018). arXiv:1808.10858.

[12] Geras, K. J., Wolfson, S., Kim, S. G., Moy, L. & Cho, K. High-resolution breast cancer screening with multi-view deep convolutional neural networks. 1–7 (2017). arXiv:1703.07047.

[13] Simonyan, K., Vedaldi, A. & Zisserman, A. Deep inside convolutional networks: visualising image classification models and saliency maps. 1–8, 10.1080/00994480.2000.10748487 (2013). arXiv:1312.6034.

[14] Nam JG (2019). Development and validation of deep learning-based automatic detection algorithm for malignant pulmonary nodules on chest radiographs. Radiology.

[15] Ronneberger, O., Fischer, P. & Brox, T. U-Net: convolutional networks for biomedical image segmentation. *MICCAI* 234–241, 10.1007/978-3-319-24574-4_28 (2015). arXiv:1505.04597.

[16] Tang, Y., Tang, Y., Xiao, J. & Summers, R. M. XLSor: a robust and accurate lung segmentor on chest X-rays using criss-cross attention and customized radiorealistic abnormalities generation. 457–467 (2019). arXiv:1904.09229.

[17] Pan I, Cadrin-Chênevert A, Cheng PM (2019). Tackling the radiological society of North America pneumonia detection challenge. Am. J. Roentgenol..

[18] Lin, T.-Y., Goyal, P., Girshick, R., He, K. & Dollár, P. Focal loss for dense object detection. 10.1016/j.ajodo.2005.02.022 (2017). arXiv:1708.02002. 10.1109/TPAMI.2018.2858826 30040631

[19] Redmon, J., Divvala, S., Girshick, R. & Farhadi, A. You only look once: unified, real-time object detection. 10.1109/CVPR.2016.91 (2015). arXiv:1506.02640.

[20] Girshick, R. Fast R-CNN. *Proceedings of the IEEE International Conference on Computer Vision* 11–18 Dec, 1440–1448, 10.1109/ICCV.2015.169 (2016). arXiv:1504.08083.

[21] Ren, S., He, K., Girshick, R. & Sun, J. Faster R-CNN: Towards real-time object detection with region proposal networks. *IEEE Trans. Pattern Anal. Mach. Intell.* **39**, 1137–1149. 10.1109/TPAMI.2016.2577031 (2017). arXiv:1506.01497. 10.1109/TPAMI.2016.2577031 27295650

[22] Kim YG (2019). Short-term reproducibility of pulmonary nodule and mass detection in chest radiographs: comparison among radiologists and four different computer-aided detections with convolutional neural net. Scientific Reports.

[23] Hwang, S. & Kim, H. E. Self-transfer learning for weakly supervised lesion localization. *Lecture Notes in Computer Science (including subseries Lecture Notes in Artificial Intelligence and Lecture Notes in Bioinformatics)* **9901 LNCS**, 239–246, 10.1007/978-3-319-46723-8_28 (2016). arXiv:1602.01625.

[24] Oquab, M., Bottou, L., Laptev, I. & Sivic, J. Is object localization for free? Weakly-supervised learning with convolutional neural networks. *Proc. IEEE Comput. Soc. Conf. Comput. Vis. Pattern Recogn.*, 685–694, 10.1109/CVPR.2015.7298668 (2015).

[25] Shapira, N. *et al.* Liver lesion localisation and classification with convolutional neural networks: a comparison between conventional and spectral computed tomography. *Biomed. Phys. Eng. Express*10.1088/2057-1976/ab6e18 (2020). 10.1088/2057-1976/ab6e18 33438626

[26] Li F, Engelmann R, Armato SG, MacMahon H (2015). Computer-aided nodule detection system: results in an unselected series of consecutive chest radiographs. Acad. Radiol..

[27] Jacobs C (2016). Computer-aided detection of pulmonary nodules: a comparative study using the public LIDC/IDRI database. Eur. Radiol..

[28] Quekel LG, Kessels AG, Goei R, Van Engelshoven JM (1999). Miss rate of lung cancer on the chest radiograph in clinical practice. Chest.

[29] Zech JR (2018). Variable generalization performance of a deep learning model to detect pneumonia in chest radiographs: a cross-sectional study. PLOS Med..

[30] Blinov, D. Advanced neural network solution for detection of lung pathology and foreign body on chest plain radiographs. 7–9.

[31] Shiraishi J (2000). Development of a digital image database for chest radiographs with and without a lung nodule. Am. J. Roentgenol..

[32] van Ginneken B, Stegmann MB, Loog M (2006). Segmentation of anatomical structures in chest radiographs using supervised methods: a comparative study on a public database. Med. Image Anal..

[33] Gaiser, H. Keras-Retinanet, Accessed 2 January 2020, 10.5281/zenodo.1188105.

[34] Abadi, M. *et al.* TensorFlow: Large-Scale Machine Learning on Heterogeneous Systems (2015).

[35] Chollet, F. *et al.* Keras, Accessed 7 December 2018 (2015).

[36] He, K., Zhang, X., Ren, S. & Sun, J. Deep Residual Learning for Image Recognition. 10.1109/CVPR.2016.90 (2015). arXiv:1512.03385.

[37] Milletari, F., Navab, N. & Ahmadi, S.-A. V-Net: Fully convolutional neural networks for volumetric medical image segmentation. *IEEE Int. Conf. 3D Vis.*, 1–11. arXiv:1606.04797 (2016).

[38] Efron B (1979). Bootstrap methods: another look at the jackknife. Ann. Stat..

